# The *PNPLA3* Ile148Met interacts with overweight and dietary intakes on fasting triglyceride levels

**DOI:** 10.1007/s12263-014-0388-4

**Published:** 2014-02-22

**Authors:** Ivana A. Stojkovic, Ulrika Ericson, Gull Rukh, Martin Riddestråle, Stefano Romeo, Marju Orho-Melander

**Affiliations:** 1 The Clinical Nutrition Unit, Department of Clinical Sciences in Malmö, Diabetes and Cardiovascular Disease, Genetic Epidemiology, Lund University, Lund, Sweden; 2 Department of Clinical Sciences, Clinical Obesity Research, Lund University, Skåne University Hospital Malmö, Malmö, Sweden; 3 Steno Diabetes Center, Gentofte, Danmark; 4 Department of Molecular and Clinical Medicine, Sahlgrenska Center for Cardiovascular and Metabolic Research, University of Gothenburg, Göteborg, Sweden; 5 Clinical Research Centre, Building 91:12, SUS in Malmö, Jan Waldenströms gata 35, 205 02 Malmö, Sweden

**Keywords:** PNPLA3, Diet, Fasting triglycerides, NAFLD, Sucrose, Polyunsaturated fatty acids

## Abstract

The Ile148Met (rs738409, G-allele) in the patatin-like phospholipase domain-containing protein 3 gene (*PNPLA3*) associates with liver fat content and may lead to loss-of-function (hydrolysis) or gain-of-function (CoA-dependent lysophosphatidic acid acyltransferase) defects. PNPLA3 is up-regulated by dietary carbohydrates, and interactions between rs738409 and carbohydrates, and sugar and ω6:ω3-polyunsaturated fatty acid (PUFA) ratio on hepatic fat accumulation have been reported. We examined interaction between rs738409 and overweight, and between rs738409 and dietary intakes (carbohydrates, sucrose and ω6:ω3-PUFA ratio), on fasting triglyceride levels. From the Malmo Diet and Cancer Study-Cardiovascular Cohort, 4,827 individuals without diabetes aged 58 ± 6 years, 2,346 with BMI ≤ 25 kg/m^2^ and 2,478 with BMI > 25 kg/m^2^, were included in cross-sectional analyses. Dietary data were collected by a modified diet history method. Overweight modified the association between rs738409 and fasting triglyceride levels (*P*_interaction_ = 0.003). G-allele associated with lower triglycerides only among overweight individuals (*P* = 0.01). Nominally, significant interaction on triglyceride levels was observed between rs738409 and sucrose among normal-weight individuals (*P*_interaction_ = 0.03). G-allele associated with lower triglycerides among overweight individuals in the lowest tertiles of carbohydrate and ω6:ω3-PUFA ratio (*P* = 0.04 and *P* = 0.001) and with higher triglycerides among normal-weight individuals in the highest tertile of sucrose (*P* = 0.001). We conclude that overweight and dietary sucrose may modify the association between rs738409 and fasting triglyceride levels.

**Electronic supplementary material:**

The online version of this article (doi:10.1007/s12263-014-0388-4) contains supplementary material, which is available to authorized users.

## Introduction

 A common missense variation Ile148Met (rs738409, G-allele), in the patatin-like phospholipase domain-containing protein 3 gene (*PNPLA3*), also named adiponutrin, was identified associated with liver fat content and non-alcoholic fatty liver disease (NAFLD) in a genome-wide association study (GWAS) in 2008 (Romeo et al. 2008). This association between the 148M (G-allele) and fatty liver and histological disease severity has since then been widely replicated in both adults and children (Kotronen et al. 2009; Kantartzis et al. 2009; Goran et al. 2010; Hyysalo et al. 2011; Santoro et al. 2010; Valenti et al. 2010; Del Giudice et al. 2011; Romeo et al. 2010a, b). Two recent studies observed that the *PNPLA3* rs738409 GG-genotype associated with lower serum triglyceride and cholesterol levels among obese (Palmer et al. 2012) or glucose-intolerant individuals (Krarup et al. 2012) and was suggested to be a result of an obesity-driven reduced triglyceride hydrolysis and intrahepatic fat accumulation (Pirazzi et al. 2012). Indeed, PNPLA3 has been shown to have a hydrolytic activity against the three major glycerolipids (Huang et al. 2011; Pignitore et al. 2013), and the PNPLA3 148M was also reported to be a loss-of-function (hydrolysis) variant with a markedly decreased *V*_max_ for glycerolipids (Huang et al. 2011). Loss of the hydrolysis function of PNPLA3 by the 148M variant has been indicated in several studies: analysis of VLDL kinetics after a bolus infusion of stable isotopes in overweight/obese men revealed that the relative secretion of VLDL1 particles was lower, the rate of hydrolysis of [^3^H]—triglycerides during lipid depletion was decreased, and the cellular accumulation of labeled triglycerides was enhanced in the presence of excess free fatty acids (FFAs) by the 148M variant (Pirazzi et al. 2012; He et al. 2010; Perttila et al. 2012).

In addition to lipase activity, both human and mice PNPLA3 were recently demonstrated to exhibit an acyl-CoA-dependent lysophosphatidic acid acyltransferase (LPAAT) activity, which promotes cellular lipid synthesis by converting LPA to phosphatidic acid (Kumari et al. 2012). High sucrose diet up-regulates liver PNPLA3 of wild-type mice, which concomitantly exhibited increased LPAAT activity. In line with this, human PNPLA3-148M had increased LPAAT activity, and gain of this function was proposed to provide a plausible biochemical mechanism for increased hepatic fat accumulation (Kumari et al. 2012).

*PNPLA3* is highly responsive to changes in energy balance. It is down-regulated by fasting and dramatically up-regulated by re-feeding (Baulande et al. 2001; Liu et al. 2004; Johansson et al. 2006; Hoekstra et al. 2010). Carbohydrate (CHO) feeding up-regulates *PNPLA3* in murine adipose tissue, and liver and glucose stimulate *PNPLA3* expression in human hepatocytes via carbohydrate response element-binding protein (ChREBP) (Perttila et al. 2012; Dubuquoy et al. 2011).

Dietary CHOs and especially sucrose intake has been proposed as a key player in liver fat deposition due to the high lipogenic potential of fructose, which stimulates hepatic de novo lipogenesis (Polson and Thompson 2003; Le et al. 2009; Maersk et al. 2012; Abdemalek et al. 2010; Sevastinova et al. 2012). In a study of 154 overweight Hispanic children, the hepatic fat content correlated positively with reported CHO and sugar intakes among children homozygous for the risk allele (GG-genotype), but not among C-allele carriers (Davis et al. 2010).

In addition to CHOs, PNPLA3 is up-regulated by certain fatty acids that inhibit its degradation (Huang et al. 2010). Moreover, dietary fat and in particular diets rich in ω-6 and low in ω-3 polyunsaturated fatty acids (PUFAs) have been suggested to contribute to metabolic defects in NAFLD (Cortez-Pinto et al. 2006). In line with this, a study in 127 children and adolescents observed a positive correlation between the ratio of ω-6 and ω-3 PUFA intakes and liver fat content among GG-genotype carriers (Santoro et al. 2012). Finally, the presence of the 148M allele in children treated with docosahexaenoic acid (22:6 ω-3) worsened the beneficial reduction induced by this fatty acid on hepatic steatosis (Nobili et al. 2013), and in obese adolescents, the oxidized fatty acids derived from linoleic acid associated with markers of liver injury only in *PNPLA3* risk-genotype carriers (Santoro et al. 2014).

In this study, we examine interaction between *PNPLA3* rs738409 and overweight on fasting blood triglyceride and serum alanine aminotransferase (ALT) levels and challenge the question if rs738409 interacts with dietary intakes of CHO, sucrose or ω-6:ω-3 PUFA ratio, on fasting triglyceride levels in adult Swedes participating the Malmö Diet and Cancer Study-Cardiovascular Cohort (MDCS-CC).

## Subjects and methods

### Study population and data collection

The Malmö Diet and Cancer Study (MDCS) is a Swedish population-based prospective cohort. During 1991–1996, all women born between 1923–1950 and men born between 1923–1945, living in the city of Malmö, were invited to participate (Berglund et al. 1993; Manjer et al. 2001). Limited Swedish language skills and mental incapacity were the only exclusion criteria. The participants filled out the questionnaires covering socioeconomic, lifestyle and dietary factors, recorded meals, and underwent a diet history interview. A total of 28,098 (40 % of the eligible persons) completed all baseline examinations (Manjer et al. 2001). From this cohort, 6,103 individuals with a baseline examination between 1991 and 1994 were randomly selected to participate in a cardiovascular cohort (MDC-CC), of whom 5,543 underwent blood sampling under standardized fasting conditions.

After an overnight fast, blood samples were drawn for the determination of serum lipids, serum insulin and whole blood glucose. Blood glucose was determined by a routine hexokinase method. Triglycerides and total cholesterol were determined on a DAX 48 automatic analyzer with use of reagents and calibrators from the supplier of the instrument (Bayer AB, Goteborg, Sweden). High-density lipoprotein (HDL) cholesterol was determined by the same procedure as used for total cholesterol but after precipitation of low-density lipoprotein (LDL) and very low-density lipoprotein (VLDL) with dextran–sulfate. LDL cholesterol was calculated from the values for triglycerides, total cholesterol and HDL cholesterol according to the Friedewald formula: LDL = total cholesterol – HDL − (triglycerides/2.2). Homeostasis model assessment for insulin resistance (HOMA-IR) was used as a measure of insulin resistance and was calculated with the following formula: insulin × blood glucose/22.5 (Nilsson et al. 2007).

From MDC-CC population (5,543), we excluded 213 individuals with prevalent type 2 diabetes, based on self-reported diabetes diagnosis or self-reported anti-diabetic regimen. After exclusion, we were left with 5,330 individuals, of whom we had information on diet, *PNPLA3* rs738409 genotype, BMI and fasting triglycerides for 4,827 individuals.

Of all MDCS participants, a subgroup of 7,198 individuals participated in another study, the Malmö Preventive Project (MPP) (Berglund et al. 1996), in which the ALT measurements were performed using standard clinical assay. MPP is a prospective cohort of southern Sweden that was set up during 1974–1992 within the Department of Medicine at Malmö University Hospital, Sweden. ALT levels were used to analyze association with rs738409, but not for interaction with diet as the ALT measurements were from an earlier time as compared to the MDCS diet collection.

The ethical committee at Lund University approved MDCS and MPP, and all the participants have given their written informed consent.

### Dietary data

Dietary intakes were collected at baseline using a combination of a 7-day menu book for recording of meals that vary from day to day (lunch and dinner meals), cold beverages and nutrient supplements, and a 168-item diet questionnaire (covering food regularly consumed during the past years) for assessment of consumption frequencies and portion sizes of regularly eaten foods that were not covered by the menu book. Finally, a 45-min interview completed the dietary assessment. The mean daily intake of foods was calculated based on frequency and portion sizes estimates from the questionnaire and menu book. The food intake was converted to energy and nutrient intakes using the MDC nutrient database where the majority of the nutrient information comes from PC-KOST2-93 from the National Food Administration in Uppsala, Sweden. The MDC method is described in detail elsewhere (Callmer et al. 1993; Wirfalt et al. 2001).

The routines for coding dietary data were slightly altered in September 1994 to shorten the interview time. This change did not reveal any major influence on the ranking of individuals (Callmer et al. 1993). The relative validity of the MDC method was evaluated in the Malmo Food study 1984–1985 (Wirfalt et al. 2001). The Pearson correlation coefficients between the reference method and the MDC method, adjusted for total energy, were in men and women 0.66/0.70 for CHO, 0.60/0.74 for the variable sugar, 0.23/0.68 for linoleic acid (ω-6), 0.55/0.44 for arachidonic acid (ω-6), 0.22/0.58 for α-linolenic acid (ω-3), 0.24/0.38 for eicosapentaenoic acid (ω-3), 0.37/0.40 for docosapentaenoic acid (ω-3), and 0.20/0.27 for docosahexaenoic acid (ω-3) (Riboli et al. 1997).

We used the following variables in this study: total energy (kcal) (including energy from fat, CHOs, protein, alcohol and fiber), CHO (E%), sucrose (E%), fat (E%), SFA (E%), MUFA (E%), PUFA (E%), P:S ratio, ω-3 PUFA, ω-6 PUFA, ω-6:ω-3 PUFA ratio, and fiber (g/1,000 kcal). The nutrient densities were calculated by dividing total nutrient intakes by non-alcohol energy intake. Tertiles were used as exposure categories of macronutrients. Individuals with potentially inaccurate reports of energy (*N* = 932) were identified as having a ratio of energy intake to the basal metabolic rate outside the 95 % CI limits of the physical activity level calculated for each individual as total energy expenditure. This procedure is described in detail elsewhere (Mattisson et al. 2005).

### Other variables used as potential confounders

Information on age was obtained from the personal identification number. Body mass index (BMI; kg/m^2^) was calculated from direct measurement of weight and height. Leisure time physical activity was assessed by asking the participants to estimate the number of minutes per week they spent on 17 different activities. The duration was multiplied by an intensity factor to create a physical activity score that was divided into tertiles and categorized as low, medium and high. Participants were classified as current smokers, ex-smokers or never smokers. Alcohol intake was classified into four categories based on grams of alcohol consumed per day: zero-consumers, low (<15 g/day in women or <20 g/day in men), medium (15–30 g/day in women or 20–40 g/day in men) and high (>30 g/day in women or >40 g/day in men). The education variables were created by classifying participants according to their highest educational level (≤8y, 9–10y, 11–13y, and university degree). Season was defined as season of diet data collection: winter (December–February), spring (March–May), summer (June–August) and fall (September–November). Diet assessment method version was defined as data collection before or after the change of coding routines in September 1994. Missing values for the variables were treated as separate categories.

### Genotyping

PNPLA3 rs738409 was genotyped using Taqman PCR method (Applied Biosystems, Foster City, CA USA) according to manufacturer’s instructions. ABI Prism Sequence Detection Systems ABI 7900HT (Applied Systems) was used for post-PCR allelic discrimination by measuring allele-specific fluorescence. Genotypes were successfully determined for 96.5 % of the individuals, and the minor allele frequency was 21 %. The genotype distribution did not deviate from Hardy–Weinberg equilibrium (*P* = 0.26), and concordance rate of repeated genotyping of all individuals was 99.9 %.

### Statistical analysis

IBM SPSS Statistics version 20.0 was used for all statistical analyses. Statistical significance was set at *P* < 0.05. We examined baseline characteristics across the *PNPLA3* genotypes for continuous variables using the general linear model (GLM) adjusted for age and sex when applicable. All continuous variables except age were Ln transformed to achieve normal distribution when testing for trend across genotypes. Associations between *PNPLA3* genotype and serum triglyceride levels in tertiles of dietary intakes were evaluated using an additive model (genotypes coded as 0, 1 or 2 indicating the number of risk G-alleles) using GLM, adjusting for age and sex in a basic model and then with additional adjustment for BMI. Interactions between *PNPLA3* genotype and tertiles of dietary intake on serum triglyceride levels were analyzed by introducing a multiplicative factor of genotype and diet tertiles (treated as continuous variables) in addition to genotype and diet to the models. The multivariate analyses were adjusted for age (continuous) and total energy intake (continuous) and the following categorical variables: sex, diet assessment method version, season, leisure time physical activity, smoking, alcohol intake and education. These covariates were identified from the literature or indicated potential confounding in the MDC cohort, due to their associations with dietary intakes. The multivariate analysis was also performed with additional adjustments for BMI (continuous) and finally also for fiber intakes. We used 25 kg/m^2^ as a limit for overweight following the statements by WHO. As earlier papers reported association between *PNPLA3* I148M and lower triglyceride levels in obese or insulin resistant individuals, i.e., 30 kg/m^2^ in Palmer et al. (2012) and a mean BMI of 28.3 kg/m^2^ in Krarup et al. (2012), we performed additional interaction analyses between *PNPLA3* genotype and severe overweight (BMI > 27.5 kg/m^2^) and obesity (BMI > 30 kg/m^2^) on triglycerides. Interaction analyses with dietary intakes were performed separately in normal-weight and overweight individuals defining normal weight as BMI ≤ 25 kg/m^2^ and overweight as BMI > 25 kg/m^2^).

## Results

### Interaction between *PNPLA3* rs738409 and obesity status on fasting triglyceride and ALT levels

The clinical characteristics and dietary intakes by the *PNPLA3* rs738409 genotypes and by overweight status (normal weight defined as BMI ≤ 25 kg/m^2^ and overweight as BMI > 25 kg/m^2^) are presented in (Table 1). Among all individuals without stratification by overweight status, the rs738409 G-allele associated significantly only with higher ALT levels (*P* = 0.005). In contrast to results in normal-weight individuals, where none of the clinical variables associated significantly with rs738409, we observed associations between the G-allele and lower triglyceride-, VLDL-cholesterol- and LDL-cholesterol levels (*P* = 0.01, *P* = 0.002 and *P* = 0.04), and with higher ALT levels (*P* = 0.001) in overweight individuals. Overweight (25 kg/m^2^ ≥ BMI > 25 kg/m^2^) modified significantly the associations between rs738409 and fasting triglyceride and ALT levels (*P*_interaction_ = 0.003 and 0.03, respectively). The interactions were similar with severe overweight (BMI > 27.5 kg/m^2^) and with obesity (BMI > 30 kg/m^2^) (*P*_interaction_ = 0.004 and 0.028, for fasting triglyceride levels, and *P*_interaction_ = 0.002 and 0.004 for ALT levels, respectively).

**Table 1:** Characteristics of the Malmö Diet and Cancer study cohort by the *PNPLA3* rs738409 genotype

	All *PNPLA3* genotype					Normal weight (BMI ≤ 25) *PNPLA3* genotype				Overweight (BMI > 25) *PNPLA3* genotype			
	*N*	CC	CG	GG	*P* trend^a^	CC	CG	GG	*P* trend	CC	CG	GG	*P* trend
*N*	4,827	3,028	1,605	194		1,465	780	101		1,561	824	93	
Age (years)	4,827	58.3	58.1	58.3	0.42	57.2	57.3	57.3	0.50	58.0	58.2	58.1	0.46
BMI (kg/m^2^)	4,824	25.7	25.7	25.8	0.57	23.0	23.0	23.0	0.12	28.4	28.5	28.0	0.87
Triglycerides (mmol/L)	4,827	1.40	1.35	1.33	0.34	1.20	1.20	1.26	0.09	1.57	1.50	1.39	0.01
VLDLl (mmol/L)	4,131	9.55	9.22	9.20	0.07	8.74	8.70	9.22	0.56	10.3	9.80	9.24	0.002
LDL (mmol/L)	4,713	4.18	4.13	4.18	0.20	4.10	4.04	4.21	0.61	4.30	4.23	4.17	0.04
HDL (mmol/L)	4,770	1.36	1.36	1.38	0.80	1.45	1.45	1.44	0.79	1.28	1.27	1.32	0.70
S-ALT (U/L)^b^	7,198	0.35	0.36	0.39	0.005	0.32	0.32	0.34	0.65	0.37	0.39	0.43	0.001
Fasting glucose (mmol/L)	4,812	5.70	5.65	5.65	0.24	5.50	5.50	5.50	0.65	5.85	5.80	5.82	0.11
Fasting insulin (pmol/L)	4,655	7.91	8.00	7.25	0.30	5.80	6.00	6.26	0.80	9.80	9.80	8.13	0.40
HOMA-IR	4,430	1.61	1.70	1.60	0.70	1.23	1.28	1.33	0.92	2.00	2.03	1.84	0.78
HbA1c (%)	4,806	4.82	4.79	4.85	0.23	4.80	4.80	4.80	0.90	4.86	4.81	4.94	0.23
Total energy intake (kcal/day)	4,827	2,349	2,350	2,347	0.82	2,453	2,419	2,426	0.22	2,337	2,356	2,298	0.84
Carbohydrate (E%)	4,827	47.0	47.0	47.0	0.54	47.0	47.2	46.5	0.92	46.6	47.2	46.5	0.12
Sucrose (E%)	4,827	8.40	8.40	8.50	0.82	8.42	8.31	7.74	0.08	8.30	8.10	8.40	0.35
Fiber (g/1,000 kcal)	4,827	9.16	9.21	9.20	0.22	9.43	9.73	9.60	0.05	9.43	9.63	9.40	0.20
Total ω-3 PUFA (E%)	4,827	0.97	0.97	0.98	0.95	0.97	0.96	1.00	0.93	1.00	1.00	1.04	0.63
Total ω-6 PUFA (E%)	4,827	4.83	4.84	4.93	0.09	4.90	4.93	5.10	0.13	4.94	4.90	4.90	0.17
Total ω-6:ω-3 PUFA ratio	4,827	5.22	5.25	5.26	0.12	5.24	5.40	5.35	0.05	5.30	5.31	5.01	0.60

General linear model adjusted for age and sex when applicable

Normal-weight and overweight individuals were set on BMI ≤ 25 and BMI > 25 kg/m^2^

^a^ *P* values are calculated from ln transformed variables when appropriate

^b^ALT levels were analyzed for 7,198 individuals of MDC cohort in another project, the Malmö Preventive Project (35)

Total energy intake and intake levels of CHO, sucrose or fiber did not significantly differ between the different rs738409 genotype carriers. The intake levels of ω-3 PUFAs, ω-6 PUFAs and the ratio of them (ω-6:ω-3 PUFA) did not differ significantly among the different genotype carriers in the whole study group nor among overweight individuals. However, in the normal-weight individuals (BMI ≤ 25 kg/m^2^), G-allele associated with somewhat higher intake levels of fiber and ω-6:ω-3 PUFA ratio (Table 1).

### Interaction analyses between *PNPLA3* rs738409 and dietary intake levels of CHO, sucrose and ω-6:ω-3 PUFA on fasting triglyceride levels

**Fig. 1:**
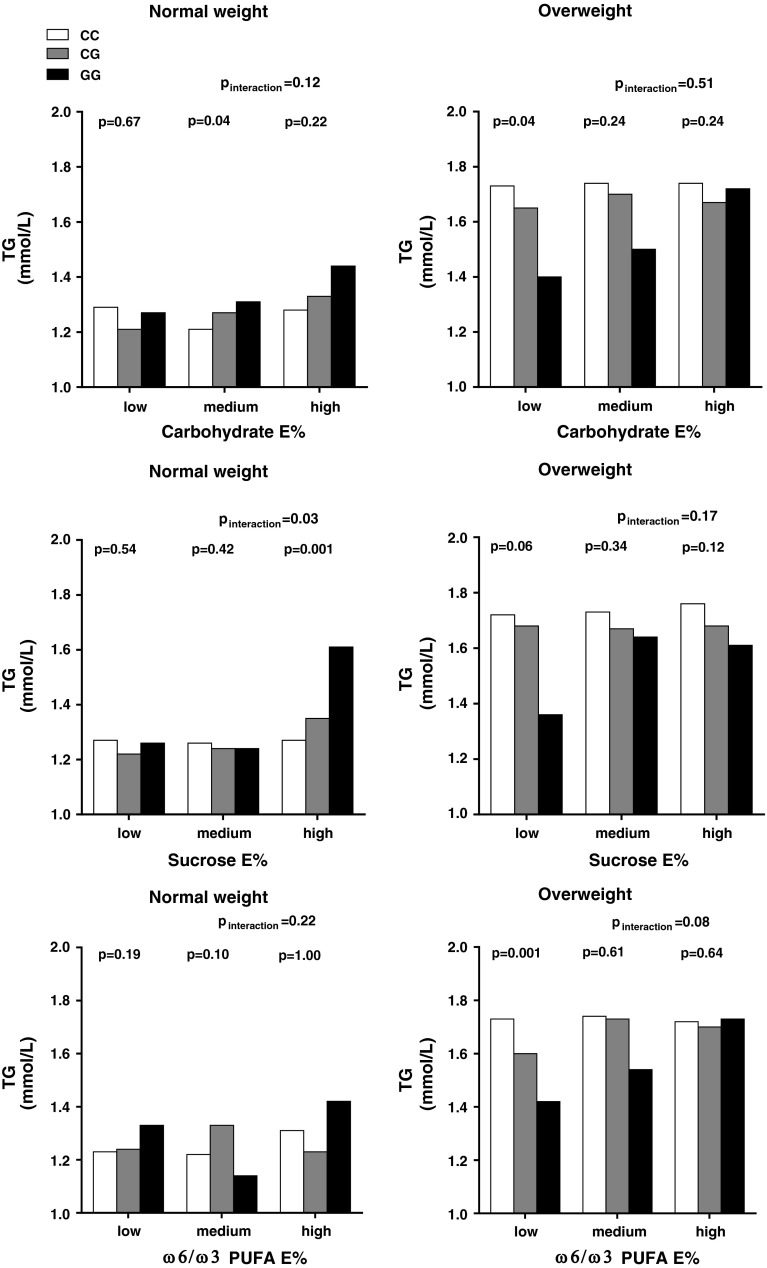
Association between the *PNPLA3* rs738409 (Ile148Met) and fasting blood triglyceride levels in strata of dietary intake categories (tertiles of CHO, sucrose and ω-6:ω-3 PUFA ratio) among individuals with normal weight (BMI ≤ 25 kg/m^2^) and overweight (BMI > 25 kg/m^2^) in the Malmö Diet and Cancer Study-Cardiovascular Cohort. Associations were calculated using general linear model and additive model for the PNPLA3 genotypes adjusting for age and sex. Interactions between genotype and dietary intakes on serum triglyceride levels were analyzed by introducing a multiplicative factor of genotype and diet tertiles (treated as continuous variables) in addition to genotype and diet to the models. Adjustments were made for age and total energy intake (as continuous variables) and sex, diet assessment method version, season, leisure time physical activity, smoking, alcohol intake and education (as categorical variables). Among normal-weight individuals, the G-allele associated with higher triglyceride levels only among those in the highest sucrose intake tertile (*P* = 0.001) but not at the lower intakes. In contrast to results in normal-weight individuals, the G-allele associated with lower triglyceride levels among overweight individuals in the lowest intake tertiles of CHO, sucrose and ω-6:ω-3 PUFA ratio (*p* = 0.04, *p* = 0.06, *p* = 0.001). The interaction between rs738409 and intakes of sucrose on triglyceride levels among normal-weight individuals was nominally significant (*P*_interaction_= 0.03)

The rs738409 G-allele associated with lower triglyceride levels in the lowest intake tertile of CHO (*P* = 0.02) in the whole study group, and this association was not seen among normal-weight individuals, but was strong among overweight individuals (Table 2). Among the overweight individuals, the association between the G-allele and lower triglycerides was only significant in the lowest intake tertiles of CHO and ω-6:ω-3 PUFA ratio (*P* = 0.04, *P* = 0.001), and similar tendency was observed in the lowest tertile of sucrose (*P* = 0.06) (Fig. 1). Among normal-weight individuals, the G-allele associated with higher triglycerides among those in the highest sucrose intake tertile (*P* = 0.001), whereas no associations were observed at the lower intakes. Statistical tests for interaction indicated a tendency for interaction between *PNPLA3* rs738409 and CHO intake (*P* = 0.07) and sucrose intake (*P* = 0.07) on triglyceride levels in the whole study population. The interaction between rs738409 and sucrose intake on triglyceride levels was nominally significant among normal-weight individuals (*P* = 0.03), while among overweight individuals, a tendency for interaction was observed with ω-6:ω-3 PUFA intake ratio (*P* = 0.08). In sensitivity analyses, after excluding 932 (19.3 %) individuals with potential inaccurate energy reporting, the interactions between *PNPLA3* rs738409 and CHO and sucrose intakes on triglyceride levels were somewhat strengthened in all individuals (*P* = 0.01 and *P* = 0.06), while the interaction with CHO intake was somewhat strengthened and with sucrose intake somewhat weakened among normal-weight individuals (*P* = 0.05 for both). The tendency for interaction with ω-6:ω-3 PUFA intake ratio in overweight individuals was weakened (*P* = 0.20), and all other observations remained virtually unchanged (see Supplementary table 2).

**Table 2:** Fasting triglyceride levels according to diet intakes, *PNPLA3* rs738409 genotype and obesity status

Triglycerides	Range	All *PNPLA3* genotype				Normal weight (BMI ≤ 25) *PNPLA3* genotype				Overweight (BMI > 25) *PNPLA3* genotype			
	Min–max	CC	CG	GG	*P* trend^a^	CC	CG	GG	*P* trend^a^	CC	CG	GG	*P* trend^a^
*Carbohydrates E*%													
*N*		3,028	1,605	194		1,465	780	101		1,561	824	93	
1st tertile	16–44	1.64 1.20, 2.11	1.54 1.10, 2.01	1.45 0.95, 1.95	0.02	1.29 1.13, 1.46	1.21 1.04, 1.38	1.27 1.02, 1.53	0.67	1.73 1.16, 2.31	1.65 1.07, 2.24	1.40 0.74, 2.05	0.04
2nd tertile	44–49	1.59 1.12, 2.10	1.61 1.14, 2.10	1.52 1.02, 2.02	0.56	1.21 1.05, 1.38	1.27 1.10, 1.44	1.31 1.05, 1.57	0.04	1.74 1.16, 2.31	1.70 1.12, 2.29	1.50 0.86, 2.13	0.24
3rd tertile	49–81	1.61 1.14, 2.10	1.60 1.13, 2.10	1.69 1.20, 2.20	0.92	1.28 1.12, 1.44	1.33 1.16, 1.50	1.44 1.17, 1.70	0.22	1.74 1.16, 2.31	1.67 1.09, 2.25	1.72 1.08, 2.36	0.24
*P* trend^b^		0.63	0.08	0.40		0.89	0.02	0.40		0.85	0.55	0.13	
(*P* interaction^b^)					(0.07)				(0.12)				(0.51)
*Sucrose E*%													
*N*		3,028	1,605	194		1,465	780	101		1,561	824	93	
1st tertile	0.60–6.70	1.61 1.15, 2.10	1.56 1.10, 2.04	1.42 0.92, 1.92	0.15	1.27 1.11, 1.43	1.22 1.05, 1.39	1.26 1.02, 1.50	0.54	1.72 1.14, 2.29	1.68 1.10, 2.27	1.36 0.71, 2.01	0.06
2nd tertile	6.70–9.31	1.61 1.14, 2.10	1.57 1.10, 2.05	1.55 1.05, 2.05	0.31	1.26 1.10, 1.42	1.24 1.07, 1.41	1.24 1.00, 1.49	0.42	1.73 1.15, 2.31	1.67 1.08, 2.25	1.64 1.01, 2.28	0.34
3rd tertile	9.31–38.0	1.62 1.15, 2.10	1.62 1.15, 2.10	1.73 1.22, 2.24	0.34	1.27 1.11, 1.43	1.35 1.18, 1.52	1.61 1.32, 1.89	0.001	1.76 1.18, 2.33	1.68 1.09, 2.26	1.61 0.96, 2.26	0.12
*P* trend^b^		0.31	0.05	0.06		0.42	0.008	0.69		0.29	0.57	0.39	
(*P* interaction^b^)					(0.07)				(0.03)				(0.17)
*ω-6:ω-3 PUFA ratio*													
*N*		3,028	1,605	194		1,465	780	101		1,561	824	93	
1st tertile	0.60–4.50	1.60 1.12, 2.10	1.52 1.04, 2.00	1.47 1.00, 2.00	0.10	1.23 1.07, 1.39	1.24 1.06, 1.40	1.33 1.07, 1.59	0.19	1.73 1.15, 2.31	1.60 1.01, 2.18	1.42 0.78, 2.05	0.001
2nd tertile	4.50–6.00	1.59 1.12, 2.10	1.64 1.20, 2.11	1.48 1.00, 2.00	0.43	1.22 1.06, 1.38	1.33 1.16, 1.50	1.14 0.83, 1.45	0.10	1.74 1.16, 2.32	1.73 1.15, 2.32	1.54 0.90, 2.18	0.61
3rd tertile	6.00–31.0	1.63 1.20, 2.10	1.56 1.10, 2.03	1.66 1.20, 2.20	0.37	1.31 1.15, 1.47	1.23 1.06, 1.39	1.42 1.19, 1.65	1.00	1.72 1.15, 2.30	1.70 1.10, 2.27	1.73 1.07, 2.38	0.64
*P* trend^b^		0.04	0.54	0.16		0.01	0.55	0.66		0.80	0.13	0.48	
(*P* interaction^b^)					(0.82)				(0.22)				(0.08)

^a^Calculations were made by using the general linear model. Basic model adjusted for age and sex

^b^Multivariate model adjusted for age, sex, diet method version, season, education, alcohol intake, smoking, total energy intake and leisure time physical activity

As we observed a tendency for interaction with the ω-6:ω-3 PUFA intake ratio among overweight individuals and as different dietary fats show a strong correlation with each other, we performed secondary interaction analyses with other fat intake variables (intakes of total fat, SFA, MUFA, PUFA, P:S ratio, ω-3 PUFA and ω-6 PUFA). However, we did not observe any significant interactions (see Supplementary table 3). Adjustments for BMI or for fiber intake did not markedly change the results. Additionally, our observations were similar in women and men (data not shown). The Pearson correlation coefficients between energy-adjusted dietary intakes are shown in Supplementary table 4.

## Discussion

We investigated the interaction between the *PNPLA3* rs738409 (Ile148Met) and overweight and dietary intakes of CHO, sucrose and ω-6:ω-3 PUFA ratio on fasting triglyceride levels. Our results indicate that the association between rs738409 and fasting triglycerides, as well as the directionality of this association, is dependent on overweight and may be modified by dietary intakes of CHO, sucrose and ω-6:ω-3 PUFA ratio. Importantly, our results suggest, using fasting triglyceride levels as a marker for liver fat accumulation, that the increased hepatic fat accumulation by 148Met may be a result of both a loss of function of the hydrolysis activity and/or a gain of function of the lipid synthase (LPAAT) activity. Moreover, although we lack the data on liver fat content, our results may suggest that overweight and dietary intakes may play a role on which of the defects of PNPLA3 148Met-mutated protein influences the phenotype.

The proportion of total variation in liver fat content attributed to rs738409 genotypes is among the strongest ever reported for common variants that associate with multifactorial phenotypes. Polymorphisms in three other genes (*GCKR*, *NCAN* and *LYPLAL1*) have been associated with liver fat accumulation and NAFLD (Speliotes et al. 2011), and the NAFLD risk variants of *GCKR* and *NCAN* associate additionally with fasting blood triglyceride levels (Speliotes et al. 2011). However, the NAFLD risk allele of *GCKR* associates with elevated, while that of *NCAN* with decreased triglyceride levels (Speliotes et al. 2011). The notion that a variant increasing the risk for hepatic fat accumulation can associate with higher or lower circulating triglyceride levels is also demonstrated by our results, suggesting that PNPLA3 148Met is capable of contributing to both lower or higher circulating triglyceride levels.

Although the PNPLA3 knock-out mice do not have any phenotype (Basantani et al. 2011; Chen et al. 2010), administration of the human mutant PNPLA3 148Met to the livers of wild-type mice increased their susceptibility of fatty liver (Huang et al. 2011). In the light of these findings and our results, it can be speculated that an increased LPAAT activity of the PNPLA3 148M produces a phenotype. In line with this, over-expression of hepatic PNPLA3 of mice markedly increased circulating triglyceride levels (Qiao et al. 2010). More recently, studies of transgenic mice over-expressing human PNPLA3 148Met provided evidence for both increased triglycerides synthesis and decreased hydrolysis of hepatic triglycerides in these animals (Li et al. 2012). Further, mice over-expressing the PNPLA3 148Met developed liver steatosis on high sucrose diet, which suggests that the PNPLA3 148Met may be involved in the hepatic de novo lipogenesis (Li et al. 2012).

Dietary sucrose up-regulates PNPLA3 in both transcriptional and posttranslational level while the PNPLA3 is virtually absent in fasted animals (Li et al. 2012; Huang et al. 2010). A high ω-6:ω-3 PUFA intake ratio has been shown to stabilize the protein, further contributing to high levels of PNPLA3 protein (Huang et al. 2010). As also high FFA levels stabilize PNPLA3, one may speculate that obese individuals, in whom FFAs in circulation are generally elevated, may have higher levels of liver PNPLA3. Without data on liver fat levels in our study, it is not easy to interpret the results of our study. We can only speculate that the reduced hydrolysis function may be reflected among overweight carriers of 148Met as lower circulating triglyceride levels, in particular, when dietary intakes of CHOs, sucrose or ω-6:ω-3 PUFA ratio were low. In such environment, the level of PNPLA3 protein can be expected to be very low, and thus, the loss of the lipase activity could be the dominating functional defect, as the importance of a partial loss of function can be expected to increase and that of any gain-of-function defect to decrease by decreasing protein levels and vice versa. In line with this, we further speculate that the reverse enzymatic turning into an increased LPAAT activity by 148Met may be reflected as higher triglyceride levels among normal-weight individuals when the level of PNPLA3 protein is expected to be high, i.e., when dietary intakes of sucrose and ω-6:ω-3 PUFA ratio were high. Two earlier studies have reported interaction between dietary intake of sugar or CHOs (Davis et al. 2010) or ω-6:ω-3 PUFA ratio and liver fat content, both in obese children or adolescents. In the first study of 154 overweight Hispanic children, a positive correlation was observed between hepatic fat content and reported CHO and sugar intake among children homozygous for 148Met (GG-genotype), but not among the other genotype carriers. Similarly, the second study of 127 children and adolescents of mainly Caucasian origin observed that the ω-6:ω-3 PUFA intake ratio correlated positively with liver fat content only among GG-genotype carriers (Santoro et al. 2012). The main finding of these studies was that the positive correlation between high intake of sugar or high ω-6:ω-3 PUFA ratio and liver fat content was restricted to individuals homozygous for 148Met (GG-genotype). Further evidence for that high ω-6: ω-3 PUFA ratio may be particularly important for liver injury among the *PNPLA3* G-allele carriers was recently provided by a study of 80 obese adolescents in which oxidized fatty acids derived from linoleic acid associated with markers of liver injury only in risk-genotype carriers (Santoro et al. 2014).

Strengths of our study include the large sample size and high-quality dietary data, based on a 7-day diet diary and large questionnaire, with information on many potential confounding factors and the ability to identify inaccurate reporters of energy intake. Still, the main limitation of our study is the lack of data on hepatic fat content. Another limitation is the lack of data on fatty acid concentrations, like those of linoleic- and alpha linolenic acids, which would have been very informative for the interpretation of the data. Further, we only had ALT levels analyzed in connection to another study almost two decades before MDCS and diet data collection, and therefore, interaction analyses between diet and ALT would not have been appropriate. On the other hand, association analyses between serum ALT levels and *PNPLA3* genotypes according to overweight status strengthened our observation of that the association between 148Met and lower triglyceride levels, putatively reflecting the hydrolysis defect, was restricted to overweight individuals and aided at the interpretation of the results of our study without information on liver fat levels. Although nutrient habits may differ between populations, our findings from this population-based study may be applicable to other westernized countries, because the nutrient intake levels could by no means be considered as extreme and are comparable with those from Swedish national surveys. Finally, we need to emphasize that we did not correct our analyses for multiple comparisons, and therefore, the nominally significant or borderline significant interactions with the dietary intakes in this study must be interpreted with caution and need to be replicated in other large studies with dietary data of good quality.

In conclusion, the findings of our epidemiological study indicate that overweight modifies the association between PNPLA3 Ile148Met on circulating triglyceride levels and suggest that dietary intakes of carbohydrates and in particular sucrose, and possibly ω-6:ω-3 PUFA ratio, may additionally modify this association. Our results are in line with the hypothesis that overweight and high sucrose intake facilitate liver fat accumulation in 148Met carriers and may suggest the defective hydrolysis function of PNPLA3-148Met to be more important in the overweight state, while the defective increase in LPAAT activity may be a more important defect when dietary intake of sucrose is high. It is thus important to challenge the clinical question of if the excess liver fat accumulation in 148Met carriers can be eliminated by weight control and reduction in dietary sucrose intake by intervention studies.

## Electronic supplementary material

Below is the link to the electronic supplementary material.

Supplementary material 1 (DOC 220 kb)

## Abbreviations

PNPLA3: Patatin-like phospholipase domain-containing protein 3
GWAS: Genome-wide association study
MDCS: Malmö Diet and Cancer Study
NAFLD: Non-alcoholic fatty liver disease
CHO: Carbohydrate
PUFA: Polyunsaturated fatty acid
ALT: Alanine aminotransferase
LPAAT: Lysophosphatidic acid acyltransferase
