# Erratum to: The *PNPLA3* Ile148Met interacts with overweight and dietary intakes on fasting triglyceride levels

**DOI:** 10.1186/s12263-016-0526-2

**Published:** 2016-04-25

**Authors:** Ivana A. Stojkovic, Ulrika Ericson, Gull Rukh, Martin Ridderstråle, Stefano Romeo, Marju Orho-Melander

**Affiliations:** 1 The Clinical Nutrition Unit, Department of Clinical Sciences in Malmö, Diabetes and Cardiovascular Disease, Genetic Epidemiology, Lund University, Lund, Sweden; 2 Department of Clinical Sciences, Clinical Obesity Research, Lund University, Skåne University Hospital Malmö, Malmö, Sweden; 3 Steno Diabetes Center, Gentofte, Danmark; 4 Department of Molecular and Clinical Medicine, Sahlgrenska Center for Cardiovascular and Metabolic Research, University of Gothenburg, Göteborg, Sweden; 5 Clinical Research Centre, Building 91:12, SUS in Malmö, Jan Waldenströms gata 35, 205 02 Malmö, Sweden

## Erratum

The author name Martin Ridderstråle was incorrectly spelt as Martin Riddestråle during the production of the original article [1]. This has now been corrected in the above author list and the publisher apologises for any inconvenience caused.
