# Supplementary material for: The PNPLA3 Ile148Met interacts with overweight and dietary intakes on fasting triglyceride levels
Source: Genes Nutr. 2014 Feb 22;9(2):388. doi: 10.1007/s12263-014-0388-4 (PMC3968290; doi:10.1007/s12263-014-0388-4)
Supplement: Supplementary file 1 — Supplementary material 1 (DOC 220 kb) [file 12263_2014_388_MOESM1_ESM.doc]

**SUPPLEMENTARY MATERIAL TO MANUSCRIPT:**

The *PNPLA3* Ile148Met interacts with obesity and dietary intakes on fasting triglyceride levels

Ivana A. Stojkovic^1^, Ulrika Ericson^1^, Gull Rukh^1^, Martin Riddestråle^2^, Stefano Romeo^3^, Marju Orho-Melander^1^

^1^Department of Clinical Sciences in Malmö, Diabetes and Cardiovascular disease, Genetic Epidemiology, Lund University, Sweden (IS, UE, GR, MO-M); the Clinical Nutrition Unit, ^2^Department of Clinical Sciences, Clinical Obesity Research, Lund University, Skåne University Hospital Malmö, Malmö, Sweden and Steno Diabetes Center, Danmark (MR), ^3^Department of Molecular and Clinical Medicine, Sahlgrenska Center for Cardiovascular and Metabolic Research, University of Gothenburg, Gothenburg, Sweden (SR).

Corresponding author E-mail address: [marju.orho-melander@med.lu.se](mailto:marju.orho-melander@med.lu.se)

**SUPPLEMENTARY TABLE 1.** Distribution of baseline lifestyle characteristics across *PNPLA3*genotypes in individuals from the Malmö Diet and Cancer Cardiovascular cohort

|  | All | | | | | Normal weight (BMI≤25)  *PNPLA3* genotype | | | | | Overweight (BMI>25)  *PNPLA3* genotype | | | | |
| --- | --- | --- | --- | --- | --- | --- | --- | --- | --- | --- | --- | --- | --- | --- | --- |
|  | n | CC | CG | GG | P-value*^a^* | n | CC | CG | GG | P-value*^a^* | n | CC | CG | GG | P-value*^a^* |
| n | 4827 | 3028 | 1605 | 194 |  | 2346 | 1465 | 780 | 101 |  | 2478 | 1561 | 824 | 93 |  |
| Alcohol intake, high (%) | 4823 | 3.8 | 3.4 | 3.1 | 0.66 | 2346 | 2.8 | 2.4 | 2.0 | 0.80 | 2474 | 4.8 | 4.3 | 4.3 | 0.82 |
| Smokers, ever (%) | 4824 | 59.4 | 60.1 | 63.0 | 0.61 | 2345 | 59.0 | 62.4 | 61.4 | 0.24 | 2476 | 60.0 | 58.0 | 64.5 | 0.39 |
| Education, low, <9 y (%) | 4820 | 46.0 | 46.0 | 42.5 | 0.65 | 2341 | 40.0 | 40.2 | 35.0 | 0.60 | 2476 | 52.0 | 51.0 | 50.5 | 0.91 |
| Leisure time physical activity, highest tertile (%) | 4796 | 33.2 | 35.0 | 34.0 | 0.58 | 2332 | 32.3 | 37.0 | 30.3 | 0.08 | 2461 | 34.0 | 33.0 | 38.0 | 0.56 |

*^a^*P-value from Chi-square test comparing the percentage of individuals between genotypes.

**SUPPLEMENTARY TABLE 2.** Sensitivity analyses*^a^* of fasting triglyceride levels and 95% CI according to diet intake and *PNPLA3* rs738409 genotype

| Triglycerides | All  *PNPLA3* genotype | | | | | Normal weight (BMI≤25)  *PNPLA3* genotype | | | | Overweight (BMI>25)  *PNPLA3* genotype | | | | | | |
| --- | --- | --- | --- | --- | --- | --- | --- | --- | --- | --- | --- | --- | --- | --- | --- | --- |
| Carbohydrates E% | CC | CG | GG | P trend*^b^* | CC | | CG | GG | P trend*^b^* | | CC | | | CG | GG | P trend*^b^* |
| N | 2459 | 1286 | 150 |  | 1240 | | 630 | 83 |  | | 1217 | | | 655 | 67 |  |
| 1^st^ tertile | 1.76  1.20,2.33 | 1.64  1.10,2.21 | 1.60  1.00,2.21 | 0.03 | 1.30  1.13,1.47 | | 1.19  1.02,1.38 | 1.32  1.05,1.59 | 0.72 | | 1.90  1.21,2.56 | | | 1.75  1.07,2.44 | 1.57  0.80,2.34 | 0.03 |
| 2^nd^ tertile | 1.72  1.15,2.30 | 1.71  1.13,2.30 | 1.64  1.04,2.25 | 0.93 | 1.24  1.10,1.41 | | 1.28  1.10,1.46 | 1.36  1.08,1.64 | 0.05 | | 1.90  1.22,2.58 | | | 1.82  1.13,2.50 | 1.61  0.86,2.36 | 0.04 |
| 3^rd^ tertile | 1.71  1.13,2.30 | 1.74  1.20,2.31 | 1.86  1.25,2.50 | 0.23 | 1.27  1.10,1.44 | | 1.34  1.17,1.52 | 1.48  1.16,1.81 | 0.08 | | 1.85  1.17,2.53 | | | 1.83  1.15,2.51 | 1.91  1.16,2.66 | 0.89 |
| P trend*^c^* | 0.25 | 0.02 | 0.57 |  | 0.72 | | 0.01 | 0.48 |  | | 0.73 | | 0.13 | | 0.12 |  |
| (P interaction*^c^*) |  |  |  | (0.01) |  | |  |  | (0.05) | |  | |  | |  | (0.17) |
| Sucrose E% | CC | CG | GG | P trend*^b^* | CC | | CG | GG | P trend*^b^* | | CC | | | CG | GG | P trend*^b^* |
| N | 2459 | 1286 | 150 |  | 1240 | | 630 | 83 |  | | 1217 | | | 655 | 67 |  |
| 1^st^ tertile | 1.75  1.20,2.33 | 1.70  1.10,2.25 | 1.56  1.00,2.20 | 0.11 | 1.28  1.12,1.45 | | 1.21  1.03,1.40 | 1.32  1.05,1.59 | 0.47 | | 1.87  1.20,2.55 | | | 1.78  1.10,2.46 | 1.42  0.65,2.19 | 0.01 |
| 2^nd^ tertile | 1.72  1.15,2.30 | 1.72  1.15,2.30 | 1.69  1.10,2.30 | 0.79 | 1.26  1.10,1.43 | | 1.26  1.10,1.44 | 1.26  1.00,1.54 | 0.67 | | 1.86  1.18,2.54 | | | 1.82  1.13,2.50 | 1.84  1.10,2.60 | 0.76 |
| 3^rd^ tertile | 1.75  1.20,2.32 | 1.74  1.20,2.31 | 1.89  1.30,2.50 | 0.33 | 1.26  1.10,1.43 | | 1.35  1.15,1.53 | 1.67  1.34,2.00 | 0.001 | | 1.90  1.13,2.58 | | | 1.81  1.13,2.49 | 1.78  1.03,2.53 | 0.11 |
| P trend*^c^* | 0.43 | 0.05 | 0.11 |  | 0.74 | | 0.02 | 1.00 |  | | 0.32 | | 0.26 | | 0.36 |  |
| (P interaction*^c^*) |  |  |  | (0.06) |  | |  |  | (0.05) | |  | |  | |  | (0.45) |
| ω-6:ω-3 PUFA ratio | CC | CG | GG | P trend*^b^* | CC | | CG | GG | P trend*^b^* | | CC | | | CG | GG | P trend*^b^* |
| N | 2459 | 1286 | 150 |  | 1240 | | 630 | 83 |  | | 1217 | | | 655 | 67 |  |
| 1^st^ tertile | 1.69  1.12,2.30 | 1.52  1.05,2.20 | 1.62  1.01,2.22 | 0.32 | 1.23  1.07,1.40 | | 1.22  1.07,1.40 | 1.42  1.12,1.71 | 0.13 | | 1.84  1.16,2.52 | | | 1.71  1.02,2.40 | 1.53  0.45,2.10 | 0.002 |
| 2^nd^ tertile | 1.70  1.13,2.30 | 1.75  1.20,2.32 | 1.63  1.00,2.25 | 0.46 | 1.23  1.07,1.40 | | 1.36  1.20,1.54 | 1.17  0.82,1.52 | 0.07 | | 1.87  1.20,2.55 | | | 1.83  1.14,2.51 | 1.71  1.00,2.47 | 0.60 |
| 3^rd^ tertile | 1.76  1.20,2.34 | 1.69  1.11,2.30 |  | 0.41 | 1.33  1.16,1.50 | | 1.24  1.06,1.42 | 1.43  1.20,1.70 | 0.90 | | 1.88  1.20,2.56 | | | 1.82  1.14,2.51 | 1.91  1.14,2.70 | 0.53 |
| P trend*^c^* | 0.006 | 0.36 | 0.22 |  | 0.007 | | 0.88 | 0.94 |  | | | 0.28 | 0.08 | | 0.82 |  |
| (P interaction*^c^*) |  |  |  | (0.80) |  | |  |  | (0.17) | | |  |  | |  | (0.20) |

*^a^*Sensitivity analysis after excluding potential inaccurate reporters of energy intake using the multivariate model.

*^b^*Calculations were made by using the general linear model. Basic model adjusted for age and sex.

*^c^*Multivariate model adjusted for age, sex, diet assessment method version, season, education, alcohol intake, smoking, total energy intake and leisure time physical activity.

**SUPPLEMENTARY TABLE 3**. Fasting triglyceride levels according to diet intakes, *PNPLA3* rs738409 genotype and obesity status

| Triglycerides | All  *PNPLA3* genotype | | | | | Normal weight (BMI≤25)  *PNPLA3* genotype | | | | | | | Overweight (BMI>25)  *PNPLA3* genotype | | | | |
| --- | --- | --- | --- | --- | --- | --- | --- | --- | --- | --- | --- | --- | --- | --- | --- | --- | --- |
| Fat E% | CC | CG | GG | P trend*^a^* | CC | | CG | | GG | | P trend*^a^* | | CC | CG | GG | | P trend*^a^* |
| N | 3028 | 1605 | 194 |  | 1240 | | 630 | | 83 | |  | | 1217 | 655 | 67 | |  |
| 1^st^ tertile | 1.62  1.15,2.10 | 1.58  1.10,2.05 | 1.65  1.14,2.15 | 0.57 | 1.27  1.11,1.43 | | 1.30  1.13,1.47 | | 1.42  1.16,1.70 | | 0.14 | | 1.75  1.17,2.33 | 1.64  1.06,2.23 | 1.70  1.03,2.33 | | 0.08 |
| 2^nd^ tertile | 1.56  1.10,2.03 | 1.61  1.10,2.04 | 1.51  1.01,2.01 | 0.22 | 1.20  1.05,1.37 | | 1.29  1.12,2.40 | | 1.29  1.01,1.56 | | 0.03 | | 1.70  1.12,2.30 | 1.70  1.12,2.30 | 1.49  0.90,2.12 | | 0.44 |
| 3^rd^ tertile | 1.63  1.16,2.10 | 1.54  1.10,2.01 | 1.48  1.00,2.00 | 0.02 | 1.31  1.15,1.48 | | 1.23  1.06,1.40 | | 1.31  1.06,1.56 | | 0.53 | | 1.73  1.16,2.31 | 1.66  1.07,2.25 | 1.46  0.80,2.12 | | 0.08 |
| P trend*^b^* | 0.70 | 0.13 | 0.54 |  | 0.38 | | | 0.07 | 0.60 |  | | 0.70 | | 0.95 | 0.12 | |  |
| (P interaction*^b^*) |  |  |  | (0.20) |  | | |  |  | (0.09) | |  | |  |  | | (1.00) |
| SFA E% | CC | CG | GG | P trend*^a^* | CC | | CG | | GG | | P trend*^a^* | | CC | CG | GG | | P trend*^a^* |
| N | 3028 | 1605 | 194 |  | 1240 | | 630 | | 83 | |  | | 1217 | 655 | 67 | |  |
| 1^st^ tertile | 1.63  1.16,2.10 | 1.60  1.12,2.10 | 1.70  1.16,2.17 | 0.90 | 1.26  1.10,1.42 | | 1.28  1.11,1.45 | | 1.50  1.23,1.78 | | 0.06 | | 1.75  1.17,2.32 | 1.70  1.10,2.26 | 1.60  1.00,2.23 | | 0.19 |
| 2^nd^ tertile | 1.63  1.16,2.10 | 1.62  1.15,2.10 | 1.56  1.10,2.06 | 0.46 | 1.27  1.11,1.43 | | 1.25  1.10,1.42 | | 1.25  1.01,1.50 | | 0.78 | | 1.75  1.17,2.33 | 1.73  1.15,2.32 | 1.66  1.02,2.30 | | 0.43 |
| 3^rd^ tertile | 1.60  1.14,2.10 | 1.56  1.10,2.03 | 1.44  0.93,1.94 | 0.22 | 1.26  1.10,1.42 | | 1.28  1.11,1.45 | | 1.29  1.03,1.55 | | 0.21 | | 1.72  1.15,2.30 | 1.63  1.04,2.21 | 1.36  0.70,2.01 | | 0.01 |
| P trend*^b^* | 0.52 | 0.27 | 0.09 |  | 1.00 | | | 1.00 | 0.93 |  | | 0.63 | | 0.26 | 0.25 | |  |
| (P interaction*^b^*) |  |  |  | (0.37) |  | | |  |  | (0.53) | |  | |  |  | | (0.49) |
| MUFA E% | CC | CG | GG | P trend*^a^* | CC | | CG | | GG | | P trend*^a^* | | CC | CG | GG | | P trend*^a^* |
| N | 3028 | 1605 | 194 |  | 1240 | | 630 | | 83 | |  | | 1217 | 655 | 67 | |  |
| 1^st^ tertile | 1.60  1.14,2.10 | 1.56  1.10,2.03 | 1.64  1.14,2.15 | 0.53 | 1.25  1.10,1.41 | | 1.29  1.12,1.46 | | 1.36  1.10,1.62 | | 0.19 | | 1.75  1.17,2.32 | 1.62  1.04,2.20 | 1.72  1.08,2.40 | | 0.06 |
| 2^nd^ tertile | 1.60  1.10,2.04 | 1.60  1.13,2.10 | 1.51  1.01,2.00 | 0.44 | 1.23  1.07,1.39 | | 1.27  1.10,1.44 | | 1.42  1.16,1.70 | | 0.02 | | 1.71  1.13,2.30 | 1.70  1.11,2.30 | 1.38  0.75,2.01 | | 0.09 |
| 3^rd^ tertile | 1.63  1.16,2.10 | 1.56  1.10,2.04 | 1.50  1.00,2.00 | 0.09 | 1.29  1.13,1.45 | | 1.22  1.05,1.39 | | 1.22  1.00,1.48 | | 0.50 | | 1.73  1.16,2.31 | 1.70  1.12,2.30 | 1.57  0.91,2.23 | | 0.49 |
| P trend*^b^* | 0.52 | 0.62 | 0.54 |  | 0.60 | | | 0.07 | 0.59 |  | | 0.77 | | 0.33 | 0.15 | |  |
| (P interaction*^b^*) |  |  |  | (0.40) |  | | |  |  | (0.13) | |  | |  |  | | (0.44) |
| PUFA E% | CC | CG | GG | P trend*^a^* | CC | | CG | | GG | | P trend*^a^* | | CC | CG | GG | | P trend*^a^* |
| N | 3028 | 1605 | 194 |  | 1240 | | 630 | | 83 | |  | | 1217 | 655 | 67 | |  |
| 1^st^ tertile | 1.60  1.10,2.04 | 1.60  1.12,2.10 | 1.60  1.10,2.10 | 0.72 | 1.25  1.10,1.40 | | 1.29  1.12,1.46 | | 1.36  1.11,1.60 | | 0.32 | | 1.71  1.14,2.30 | 1.68  1.11,2.26 | 1.62  1.00,2.30 | | 0.67 |
| 2^nd^ tertile | 1.61  1.14,2.10 | 1.56  1.10,2.03 | 1.60  1.10,2.10 | 0.41 | 1.22  1.06,1.38 | | 1.28  1.11,1.45 | | 1.40  1.12,1.70 | | 0.007 | | 1.76  1.18,2.35 | 1.64  1.06,2.23 | 1.53  0.90,2.17 | | 0.003 |
| 3^rd^ tertile | 1.65  1.20,2.11 | 1.60  1.12,2.10 | 1.51  1.00,2.00 | 0.28 | 1.32  1.15,1.48 | | 1.23  1.06,1.40 | | 1.26  1.01,1.52 | | 0.69 | | 1.76  1.18,2.34 | 1.74  1.16,2.33 | 1.55  0.91,2.18 | | 0.41 |
| P trend*^b^* | 0.04 | 0.50 | 0.50 |  | 0.36 | | | 0.06 | 0.33 |  | | 0.18 | | 0.37 | 0.44 | |  |
| (P interaction*^b^*) |  |  |  | (0.17) |  | | |  |  | (0.16) | |  | |  |  | | (0.81) |
| ω-3 PUFA E% | CC | CG | GG | P trend*^a^* | CC | | CG | | GG | | P trend*^a^* | | CC | CG | GG | | P trend*^a^* |
| N | 3028 | 1605 | 194 |  | 1240 | | 630 | | 83 | |  | | 1217 | 655 | 67 | |  |
| 1^st^ tertile | 1.60  1.12,2.10 | 1.61  1.14,2.10 | 1.70  1.20,2.20 | 0.32 | 1.27  1.10,1.43 | | 1.29  0.50,2.00 | | 1.42  0.60,2.11 | | 0.33 | | 1.70  1.13,2.30 | 1.72  1.14,2.31 | 1.77  1.12,2.42 | | 0.47 |
| 2^nd^ tertile | 1.61  1.14,2.10 | 1.54  1.10,2.02 | 1.54  1.03,2.05 | 0.12 | 1.24  1.10,1.40 | | 1.17  0.42,1.91 | | 1.24  0.50,2.01 | | 0.36 | | 1.79  1.21,2.37 | 1.64  1.06,2.22 | 1.52  0.90,2.17 | | 0.004 |
| 3^rd^ tertile | 1.61  1.14,2.10 | 1.57  1.10,2.04 | 1.44  0.94,2.00 | 0.24 | 1.25  1.10,1.41 | | 1.11  0.50,2.00 | | 1.11  0.35,1.90 | | 0.41 | | 1.74  1.17,2.32 | 1.69  1.11,2.28 | 1.44  0.81,2.07 | | 0.04 |
| P trend*^b^* | 0.74 | 0.16 | 0.43 |  | 0.20 | | | 0.18 | 1.00 |  | | 0.61 | | 0.49 | 0.13 | |  |
| (P interaction*^b^*) |  |  |  | (0.12) |  | | |  |  | (0.75) | |  | |  |  | | (0.11) |
| ω-6 PUFA E% | CC | CG | GG | P trend*^a^* | CC | | CG | | GG | | P trend*^a^* | | CC | CG | | GG | P trend*^a^* |
| N | 3028 | 1605 | 194 |  | 1240 | | 630 | | 83 | |  | | 1217 | 655 | | 67 |  |
| 1^st^ tertile | 1.60  1.12,2.10 | 1.60  1.11,2.10 | 1.61  1.10,2.11 | 1.00 | 1.25  1.10,1.41 | | 1.29  1.11,1.46 | | 1.41  1.15,1.67 | | 0.27 | | 1.71  1.14,2.30 | 1.65  1.10,2.45 | | 1.57  0.70,2.35 | 0.20 |
| 2^nd^ tertile | 1.62  1.15,2.10 | 1.60  1.12,2.10 | 1.45  0.95,2.00 | 0.36 | 1.23  1.07,1.40 | | 1.31  1.14,1.50 | | 1.23  1.00,1.49 | | 0.02 | | 1.76  1.18,2.34 | 1.67  1.10,2.45 | | 1.44  0.74,2.25 | 0.01 |
| 3^rd^ tertile | 1.66  1.20,2.13 | 1.60  1.13,2.10 | 1.63  1.13,2.13 | 0.48 | 1.33  1.17,1.49 | | 1.24  1.07,1.41 | | 1.40  1.12,1.63 | | 0.91 | | 1.76  1.18,2.34 | 1.73  1.20,2.53 | | 1.69  1.22,3.00 | 0.58 |
| P trend*^b^* | 0.04 | 0.76 | 0.23 |  | 0.27 | | | 0.12 | 0.35 | |  | | 0.22 | 0.27 | 0.79 | |  |
| (P interaction*^b^*) |  |  |  | (0.41) |  | | |  |  | | (0.23) | |  |  |  | | (0.65) |
| P:S ratio | CC | CG | GG | P trend*^a^* | CC | | CG | | GG | | P trend*^a^* | | CC | CG | | GG | P trend*^a^* |
| N | 3028 | 1605 | 194 |  | 1240 | | 630 | | 83 | |  | | 1217 | 655 | | 67 |  |
| 1^st^ tertile | 1.56  1.11,2.05 | 1.60  1.13,2.10 | 1.51  1.01,2.01 | 0.74 | 1.24  1.10,1.40 | | 1.32  1.15,1.50 | | 1.28  1.03,1.54 | | 0.12 | | 1.71  1.13,2.30 | 1.65  1.07,2.24 | | 1.51  0.85,2.17 | 0.29 |
| 2^nd^ tertile | 1.62  1.15,2.10 | 1.55  1.10,2.03 | 1.60  1.10,2.10 | 0.15 | 1.24  1.10,1.40 | | 1.24  1.07,1.41 | | 1.38  1.11,1.65 | | 0.23 | | 1.75  1.17,2.32 | 1.64  1.05,2.22 | | 1.58  0.94,2.22 | 0.01 |
| 3^rd^ tertile | 1.66  1.20,2.13 | 1.62  1.15,2.10 | 1.60  1.10,2.07 | 0.60 | 1.31  1.15,1.50 | | 1.26  1.10,1.43 | | 1.36  1.11,1.62 | | 0.78 | | 1.76  1.18,2.34 | 1.74  1.16,2.33 | | 1.56  0.92,2.19 | 0.45 |
| P trend*^b^* | 0.03 | 0.83 | 0.32 |  | 0.20 | | | 0.09 | 0.51 | |  | | 0.17 | 0.10 | 1.00 | |  |
| (P interaction*^b^*) |  |  |  | (0.41) |  | | |  |  | | (0.31) | |  |  |  | | (0.88) |

*^a^*Calculations were made by using the general linear model. Basic model adjusted for age and sex.

*^b^*Multivariate model adjusted for age, sex, diet method version, season, education, alcohol intake, smoking, total energy intake and leisure time physical activity.

**SUPPLEMENTARY TABLE 4.** Pearson correlations coefficients*^a^* between energy adjusted intake of carbohydrates, sucrose, fat, SFA, MUFA, PUFA, P:S ratio, ω-3, ω-6 and ω-6:ω-3 PUFA ratio

|  | Carbohydrates | Sucrose | Fat | SFA | MUFA | PUFA | P:S ratio | ω-3 PUFA | ω-6 PUFA | ω-6:ω-3 PUFA ratio |
| --- | --- | --- | --- | --- | --- | --- | --- | --- | --- | --- |
| Carbohydrates | 1.0 | 0.40 | -0.92 | -0.77 | -0.85 | -0.39 | 0.25 | -0.44 | -0.34 | 0.12 |
| Sucrose | 0.40 | 1.0 | -0.22 | -0.11 | -0.21 | -0.23 | -0.10 | -0.21 | -0.20 | -0.002 |
| Fat | -0.92 | -0.22 | 1.0 | 0.86 | 0.92 | 0.42 | -0.28 | 0.42 | 0.38 | -0.08 |
| SFA | -0.77 | -0.11 | 0.86 | 1.0 | 0.67 | -0.02 | -0.65 | 0.18 | -0.05 | -0.25 |
| MUFA | -0.85 | -0.21 | 0.92 | 0.67 | 1.0 | 0.46 | -0.16 | 0.40 | 0.41 | -0.04 |
| PUFA | -0.39 | -0.23 | 0.42 | -0.02 | 0.46 | 1.0 | 0.70 | 0.56 | 0.97 | 0.37 |
| P:S ratio | 0.25 | -0.10 | -0.28 | -0.65 | -0.16 | 0.70 | 1.0 | 0.28 | 0.70 | 0.42 |
| ω-3 PUFA | -0.44 | -0.21 | 0.42 | 0.18 | 0.40 | 0.56 | 0.28 | 1.0 | 0.38 | -0.50 |
| ω-6 PUFA | -0.34 | -0.20 | 0.38 | -0.05 | 0.41 | 0.97 | 0.70 | 0.38 | 1.0 | 0.53 |
| ω-6:ω-3 PUFA ratio | 0.12 | -0.002 | -0.08 | -0.25 | -0.04 | 0.37 | 0.42 | -0.50 | 0.53 | 1.0 |

*^a^*P<0.001 for all correlations, except NS correlation between energy adjusted intake of sucrose and ω-6:ω-3 PUFA ratio, and SFA and PUFA
